# A non-invasive method for screening mitochondrial diabetes

**DOI:** 10.3389/fgene.2025.1536331

**Published:** 2025-05-30

**Authors:** Hangyu Fang, Xiaoe Li, Shuping Wang, Mei Zhang, Victor Wei Zhang, Chao Xu

**Affiliations:** ^1^ Department of Endocrinology, Shandong Provincial Hospital Affiliated to Shandong First Medical University, Jinan, Shandong, China; ^2^ Key Laboratory of Endocrine Glucose & Lipids Metabolism and Brain Aging, Ministry of Education, Jinan, Shandong, China; ^3^ Department of Endocrinology, Weishan County People’s Hospital, Jining, Shandong, China; ^4^ Department of Endocrinology and Metabolism, Dongying People’s Hospital, Dongying, Shandong, China; ^5^ Affiliated Hospital of Jining Medical University, Jining, Shandong, China; ^6^ Department of Genomic Medicine, AmCare Genomics Lab, Guangzhou, Guangdong Province, China; ^7^ Shandong Key Laboratory of Endocrinology and Lipid Metabolism, Jinan, Shandong, China; ^8^ Shandong Institute of Endocrine and Metabolic Diseases, Jinan, Shandong, China; ^9^ “Chuangxin China” Innovation Base of Stem Cell and Gene Therapy for Endocrine Metabolic Diseases, Jinan, Shandong, China; ^10^ Shandong Engineering Laboratory of Prevention and Control for Endocrine and Metabolic Diseases, Jinan, Shandong, China; ^11^ Shandong Engineering Research Center of Stem Cell and Gene Therapy for Endocrine and Metabolic Diseases, Jinan, Shandong, China

**Keywords:** mitochondrial diabetes mellitus, m.3243A>G, mutations, screening, non-invasive

## Abstract

**Background:**

Mitochondrial diabetes mellitus (MDM) is a special type of diabetes resulting from functional defects in mitochondria. Its incidence rate is low, and it can often be misdiagnosed as either type 1 or type 2 diabetes in clinical settings. Due to limited clinical experience in diagnosing and treating MDM, the rate of missed diagnosis is high. Therefore, employing appropriate detection methods for the rapid screening of suspected MDM patients can facilitate early diagnosis of MDM.

**Methods:**

We conducted a multicenter observational study by collecting oral exfoliated cells from patients and detecting the m.3243A>G mutation using Polymerase Chain Reaction (PCR). We estimated the positivity rate of MDM and clinically evaluated the detection method through clinical trials. Additionally, we summarized the clinical phenotypes of patients who tested positive and compared the clinical manifestations between MDM and non-MDM patients using statistical analysis, providing a diagnostic foundation for clinicians.

**Results:**

We collected data from a total of 478 patients and identified 16 cases of m.3243A>G mutation-positive patients by collecting oral exfoliated cell samples for PCR testing, yielding a positivity rate of 3.35% and an asymptomatic carrier rate of 0.84%. These results are slightly higher than those reported in previous research. The gene mutation detection method demonstrated high credibility and was non-invasive, with a clinical sensitivity of 87.2% and clinical specificity of 96.9%. Additionally, patient satisfaction was high in this study. Statistical analysis revealed a significant difference in clinical manifestations between MDM and non-MDM patients. MDM patients were more likely to experience neurological hearing loss and multiple systemic manifestations, and their condition was consistent with maternal inheritance, in line with previous research findings.

**Conclusion:**

The detection of the m.3243A>G mutation through the collection of oral exfoliated cells offers several advantages over other methods, including simplicity, non-invasiveness, and high specificity and sensitivity. However, it is currently underutilized. Therefore, further experiments are needed to study and validate this approach in order to optimize MDM screening methods and improve diagnostic rates for MDM.

## 1 Introduction

The prevalence of mitochondrial diseases ranges between 11% and 15%, and these conditions can lead to a wide array of endocrine complications, with diabetes being the most prominent. Mitochondrial diabetes mellitus (MDM) is a rare form of monogenic diabetes, occurring in approximately 1% of diabetes cases (Ding et al., 2022). It is associated with deletions (Ballinger et al., 1992), insertions, or point mutations in mitochondrial DNA (mtDNA) (You et al., 2022). The m.3243A>G mutation is the most common mutation site in MDM, accounting for approximately 85% of all MDM mutations (Maassen et al., 2005). The clinical phenotypes of MDM and Maturity-Onset Diabetes of the Young (MODY) are similar. Both are characterized by early-onset diabetes, non-insulin dependence in the early stages, and a lack of typical features of type 1 diabetes (such as acute onset, significant ketosis tendency, and positive autoantibodies). Both also exhibit familial inheritance. However, the two differ in that MDM demonstrates maternal inheritance, while MODY follows an autosomal dominant inheritance pattern. MDM patients may present with additional systemic symptoms such as hearing loss, central nervous system (CNS) abnormalities, skeletal muscle issues, cardiomyopathy, retinitis pigmentosa (RP), and external ophthalmoplegia (Meas et al., 2010), whereas MODY typically lacks other systemic manifestations. Over time, MDM is associated with a gradual decline in pancreatic β-cell function, and it may also be linked to a low body mass index (BMI) and negative pancreatic autoantibody tests. As a result, some MDM patients may eventually develop insulin-dependent diabetes. In contrast, MODY generally does not require insulin treatment (especially GCK-MODY), although some subtypes (such as HNF1A-MODY) may necessitate insulin therapy as the condition progresses. Studies indicate that the prevalence of MDM increases with age, and the average age at diagnosis typically ranges from 32 to 38 years. Globally, MDM accounts for approximately 3% of all diabetes cases. In China, MDM caused by mitochondrial gene mutations is estimated to affect 0.6%–1.8% of diabetic patients. However, due to limited awareness and understanding of MDM, many cases are at risk of misdiagnosis or being overlooked (Naing et al., 2014). As a result, the actual incidence of MDM may be significantly higher than the currently reported statistics suggest.

Currently, several methods are available for detecting mitochondrial gene mutations, including Sanger sequencing and second-generation sequencing. However, some hospitals prefer mitochondrial functional testing over genetic testing, leading to a lack of uniformity in clinical testing standards. Both domestically and internationally, blood samples from patients are primarily used to screen for the m.3243A>G mutation site associated with mitochondrial diabetes. However, this approach has several limitations, such as the need for invasive sampling, relatively high costs, and complex operational procedures, making it unsuitable for large-scale population screening (Wei et al., 2016). Additionally, Studies have shown that, compared to fibroblasts, skeletal muscle cells, or epithelial cells obtained from mouthwash samples, the mutation load of mitochondrial DNA (mtDNA) in blood cells of some patients is relatively low, approaching the limit of detectability. As a result, it is recommended to prioritize the screening for heteroplasmic m.3243 mutations on mtDNA using cell types other than blood cells (Wei et al., 2016; Gerbitz et al., 1995).

In this study, we utilized a non-invasive screening method to detect the m.3243A>G mutation in diabetes patients. This approach offers several advantages, including ease of sampling, absence of trauma during collection, and high patient acceptability. Additionally, we performed a statistical analysis to compare the clinical characteristics of patients with and without the mutation. Our research preliminarily validates a novel non-invasive method for mtDNA mutation screening and summarizes the clinical features of mtDNA-related disorders, providing valuable guidance for clinicians in the diagnosis of these conditions.

## 2 Object and method

### 2.1 Ethical approval

The study described herein was granted approval by the Ethics Committee of the Affiliated Provincial Hospital of Shandong First Medical University. The research protocol adheres to the ethical principles outlined in the revised Helsinki Declaration (2013) from Brazil. Informed consent was obtained from all individual participants involved in the study, with written documentation of consent being secured prior to their inclusion in the research.

### 2.2 Patients

We enrolled 478 patients with diabetes, as well as individuals with prediabetes and those with a family history of diabetes. Most of these participants were under the age of 40, and they included individuals with type 1, type 2, and other forms of diabetes.

Inclusion criteria: to investigate the clinical data of the patients treated, and meet any of the following inclusion criteria: subjects with clinically diagnosed diabetes (including type I, type II and other types of diabetes, regardless of gender and age); People with pre diabetes and diabetes family history. Exclusion criteria: those who have one or more of the following conditions: clinically diagnosed as non-diabetes patients; The sample quality does not meet the operational requirements; Repeated inclusion, not meeting the inclusion criteria.

### 2.3 Sample and clinical information collection

We collected oral exfoliated cells from patients with diabetes, individuals with prediabetes, and those with a family history of diabetes who sought healthcare at multiple institutions in Shandong Province between 2022 and the end of November 2023. These institutions included Shandong Provincial Hospital, Shandong Qianfo Mountain Hospital, Yantai Affiliated Hospital of Binzhou Medical University, Jining Affiliated Hospital, the Second Affiliated Hospital of Shandong First Medical University, Zaozhuang Municipal Hospital, Weihai Municipal Hospital, Tai’an Central Hospital, Dongying People’s Hospital, Linyi People’s Hospital, Weishan County People’s Hospital, Yanzhou District People’s Hospital, Yantai Yuhuangding Hospital, and Qingdao University Affiliated Hospital. Additionally, we collected comprehensive clinical data, including patients’ age (current age and age at onset), gender, BMI, family history, disease duration, treatment methods, islet-related antibodies, islet function, and multisystem clinical manifestations such as hearing test results, central nervous system abnormalities (e.g., epilepsy, stroke-like episodes, migraines), myopathy, lactic acidosis, and diabetes-related complications.

Patients screening positive for the m.3243A>G mutation were classified as the mutation-positive group, while the remaining patients were classified as the mutation-negative group. The clinical characteristics of patients in both the mutation-positive and mutation-negative groups were then compared. The Expert Consensus on Clinical Laboratory Diagnosis of Mitochondrial Diabetes Mellitus states that patients with a score ≥4 points are considered high-risk for mitochondrial diabetes mellitus (MDM). The scoring criteria are as follows: Age of onset <40 years in patients with type 2 diabetes mellitus (1 point); Non-obese body type in patients with type 2 diabetes mellitus (1 point); Negative pancreatic-related antibody testing (1 point); Presence of sensorineural hearing impairment (1 point); Presence of other multi-system clinical manifestations (2 points); Short disease duration with progressive decline in pancreatic β-cell secretory function during the course, rapid failure of oral medications requiring insulin therapy (2 points); Familial transmission of diabetes consistent with maternal inheritance (3 points) (中国医师协会检验医师分会线粒体疾病检验医学专家委员会, 2021). Patients in this study were scored according to the aforementioned criteria. Those with a score of ≥4 points were classified as high-risk for MDM, while those with a score of <4 points were classified as low-risk for MDM.

### 2.4 Sample testing

Insert the head of a sterile swab into the oral cavity and gently scrape the inside of the cheek to collect oral exfoliated cells. After sampling, place the swab head into a centrifuge tube containing 1 mL of sterile physiological saline. Break the swab at the crease near the swab head, leaving the head in the tube, and securely close the centrifuge tube cap. Vortex or shake the tube for 1 min to ensure thorough washing of the exfoliated cells from the swab head. Finally, discard the swab after the cells have been released into the saline solution.

Take at least 200 µL of the oral exfoliated cell suspension and centrifuge it at 12,000 rpm for 10 min. Discard the supernatant and add 50 µL of DNA extraction buffer and 5 µL of proteinase K to resuspend the cell pellet. Mix thoroughly and heat the mixture at 95°C–100°C for 10 min. Centrifuge again at 12,000 rpm for 10 min, then carefully transfer 50 µL of the supernatant into a 1.5 mL centrifuge tube, ensuring no precipitate is transferred. This supernatant contains the extracted DNA. Measure the DNA concentration and purity using a NanoDrop spectrophotometer. For Real-Time Amplification Refractory Mutation System Quantitative Polymerase Chain Reaction (ARMS qPCR), specific primers and probes are used to distinguish between wild-type and mutant alleles. Each reaction mixture consists of 18 µL of PCR master mix and 2 µL of DNA template, making a total volume of 20 µL. The thermal cycling conditions are as follows: a 2-min Uracil-N-glycosylase (UNG) enzyme reaction at 37°C, activation of the Taq enzyme at 95°C for 30 s, followed by 40 cycles of 10 s at 95°C (denaturation) and 30 s at 58°C (annealing, elongation, and fluorescence collection). Each run includes a no-template control and positive controls with known mutation levels to ensure accuracy. After the qPCR instrument completes the run, use the accompanying software to analyze the experimental results.

**FIGURE 1:**
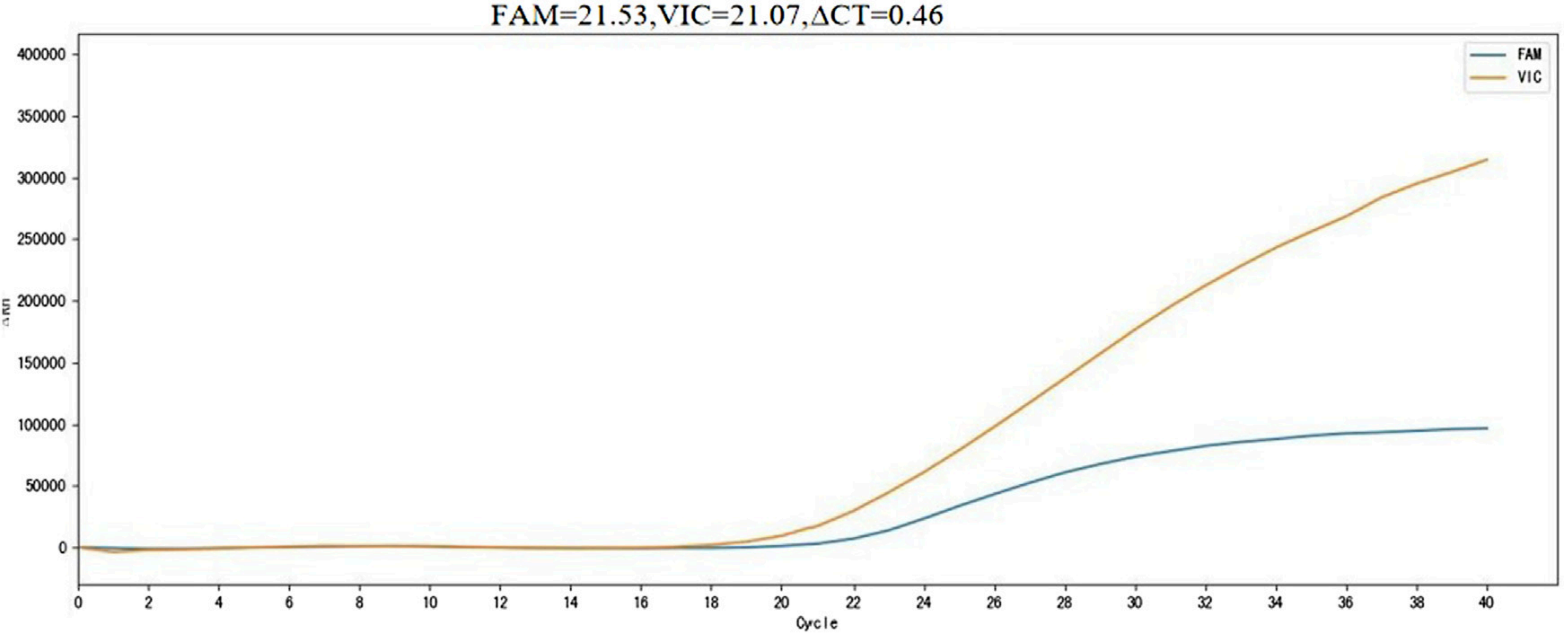
m. 3243A>G mutation positive sample detection results. FAM, m.3243A>G mutation site channel; VIC, Internal reference gene channel; ∆CT, Cut off value; Horizontal axis, number of cycles; Vertical axis, Fluorescence value.

If the fluorescein (FAM) channel shows a cycle threshold (Ct) value and a clear amplification curve, and the virtual instrumentation console (VIC) channel has a Ct value with ΔCt ≤ 14.8, the detection result is considered positive for the m.3243A>G mutation. If ΔCt > 14.8, the result is considered negative for the m.3243A>G mutation. The positive threshold for the kit was determined using the receiver operating characteristic (ROC) curve method, with a cut-off value of ΔCt = 14.8 (Figure 1).

### 2.5 Statistic analysis

Use SPSS 27.0 statistical software for data analysis. The difference in clinical characteristics between the two groups of patients was analyzed using the corrected chi square test and Fisher’s test, with *P* < 0.05 indicating statistical significance.

### 2.6 Literature collection

Pubmed takes “mitochondrial diabetes or MDM” as the keyword (http://www.pubmed.gov) search for relevant case reports to collect information on carriers of m.3243A>G.

## 3 Results

### 3.1 Positive rate

**FIGURE 2:**
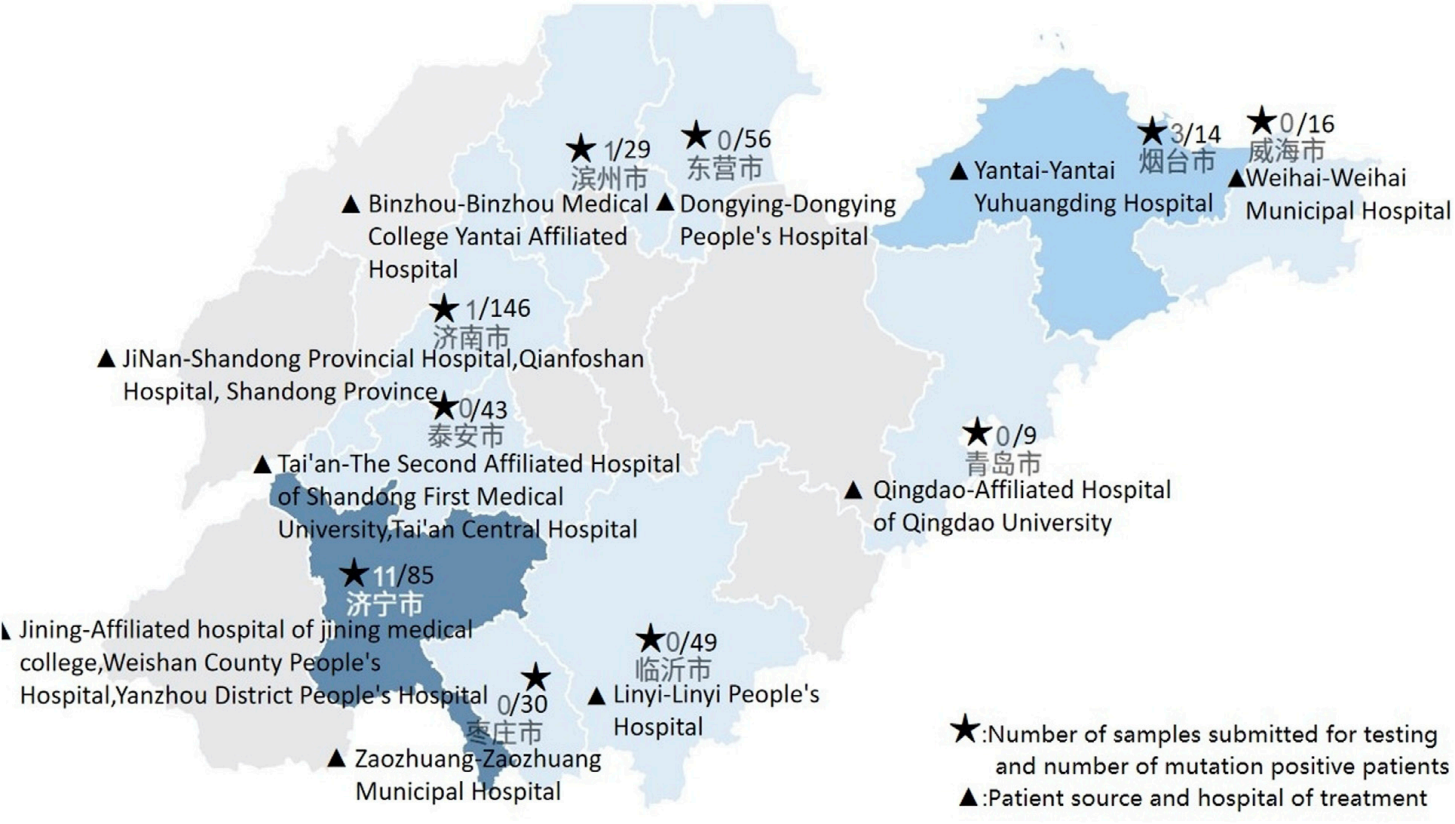
Sample submission status of each hospital.

From 2022 to the end of November 2023, a total of 2,025 samples were tested nationwide, with 73 positive cases, yielding a positive rate of 3.60%. After excluding related family samples, that is, removing other positive patients from the proband’s family, the positive rate was 2.74%. In this multicenter study, 478 patient samples were tested, of which 16 were positive, resulting in a positivity rate of 3.35%. After excluding family-related samples, the positivity rate was 2.33%. Notably, Weishan County People’s Hospital and Yantai Yuhuangding Hospital exhibited abnormally high positive rates. These rates are all higher than the proportion reported in the expert consensus (Figure 2).

### 3.2 Differences in clinical manifestations

We followed up and collected clinical information from 16 positive patients and 100 negative patients. Due to incomplete or missing patient information, we ultimately obtained clinical information from nine positive patients and 71 negative patients (Table 1). The chi square analysis of the clinical manifestations of the patients in the mutation positive group and the negative group was carried out by SPSS software. The results showed that there were statistically significant differences between the two groups in terms of whether they had neurological hearing impairment, whether they had clinical manifestations of multiple systems, whether they were maternal inheritance, and whether they had diabetes ketoacidosis (*P* < 0.05).There was no statistically significant difference in the age of onset, body type, insulin related antibody status, and duration of disease between the two groups of patients, with *P* values > 0.05 (Table 2).

**TABLE 1:** Composition of patient samples.

Gender	Number of patients (cases)	Number of diabetic patients (cases)	Number of patients with m.3243A>G mutation (cases)	Median age of onset	Age of onset (Q1, Q3)	Number of patients with a family history of diabetes (cases)	Number of matrilineal inheritance (cases)
Male	54	54	6	30	(24, 35)	34	6
Female	26	26	3	29	(22, 33)	14	3

**TABLE 2:** Comparison of clinical manifestations between the positive and negative groups of m.3243A>G mutation.

a. Clinical features	m. 3243A>G mutation positive patients (cases)	m. 3243A>G mutation negative patients (cases)	Chi square value	*P*-value
Age of onset ≤ 40	9	61	0.343	0.558
Non obese body type	8	48	0.763	0.383
Insulin related antibody negative	9	56	1.031	0.310
Accompanied by neurogenic hearing loss	4	1	—	<0.001
Clinical manifestations of multiple systems^a^	6	4	21.568	<0.001
Short course of illness^b^	4	23	0.100	0.752
Consistent with maternal inheritance	9	0	69.404	<0.001
Diabetic ketoacidosis	0	32	5.149	0.023

b. Mutation	Age		Total
	≤40 years old (cases)	>40 years old (cases)	
Positive	9	0	9
Negative	61	9	70
Total	70	9	79

c. Mutation	Body type		Total
	Non - obese (cases)	Obese (cases)	
Positive	8	1	9
Negative	48	22	70
Total	56	23	79

d. Mutation	Insulin - related antibodies		Total
	Negative (cases)	Positive (cases)	
Positive	9	0	9
Negative	56	14	70
Total	65	14	79

e. Mutation	Neurological hearing impairment		Total
	Present (cases)	Absent (cases)	
Positive	4	5	9
Negative	1	69	70
Total	5	74	79

f. Mutation	Multisystem clinical manifestations		Total
	Present (cases)	Absent (cases)	
Positive	6	3	9
Negative	4	66	70
Total	10	69	79

g. Mutation	Diabetic ketoacidosis		Total
	Present (cases)	Absent (cases)	
Positive	0	0	9
Negative	32	38	70
Total	32	38	79

h. Mutation	Consistent with matrilineal inheritance		Total
	Yes (cases)	No (cases)	
Positive	9	0	9
Negative	0	70	70
Total	9	70	79

i. Mutation	Short disease course		Total
	Yes (cases)	No (cases)	
Positive	4	5	9
Negative	23	47	70
Total	27	52	79

^a^ Multisystem clinical manifestations include impairments in the central nervous system, cardiac system, skeletal muscle system, retinal system, and others.

^b^ A short course of disease refers to the situation where oral medications become ineffective quickly and insulin therapy is required.

### 3.3 Establishment of family chart

**FIGURE 3:**
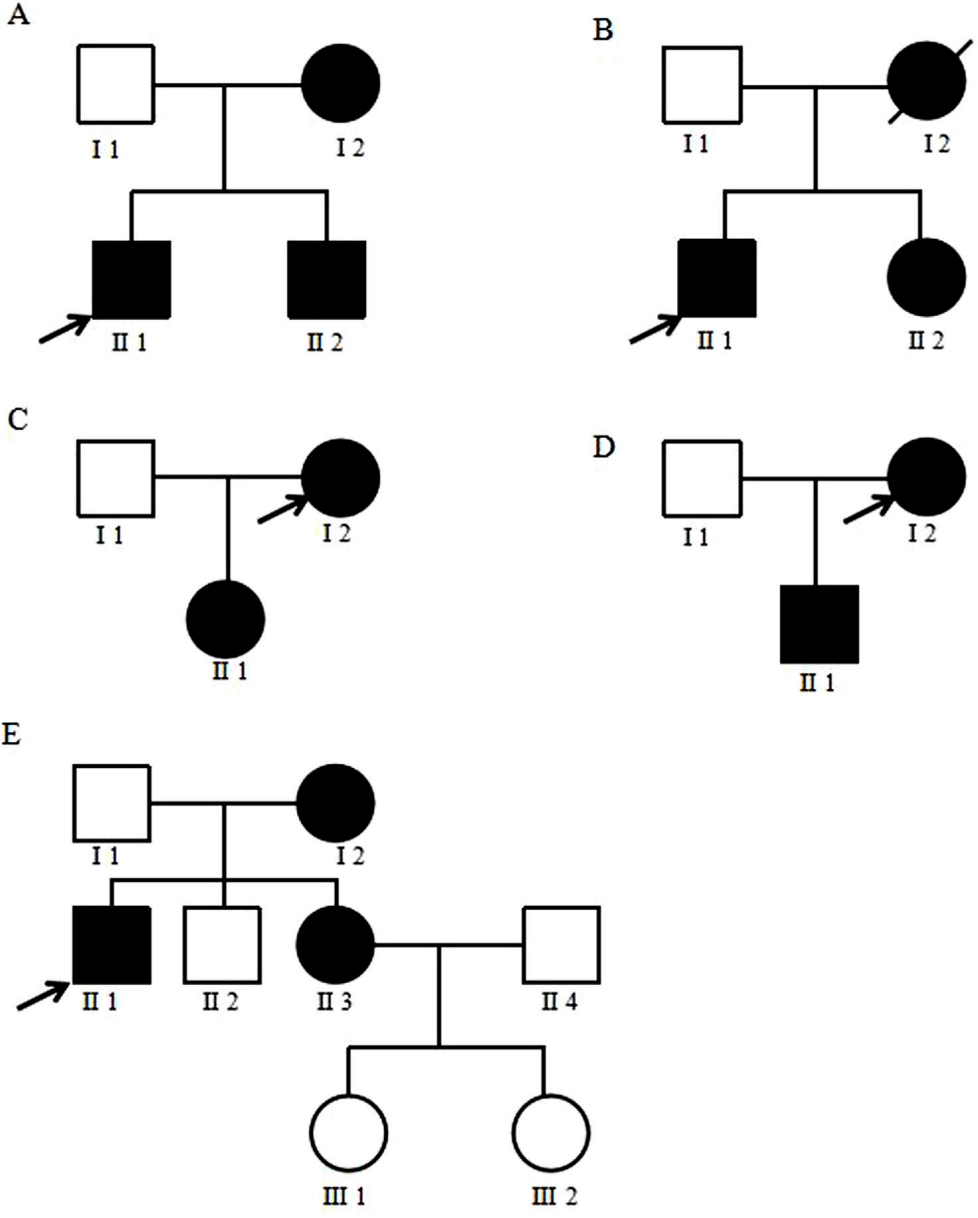
Family diagram of mitochondrial diabetes. **(A–E)** represents five mitochondrial diabetes families. The proband is indicated by a black arrow. Black refers to patients with genetic mutations, blank refer to individuals completely unaffected by the mutation, and slash refers to patients who have passed away.

The pedigree map of mitochondrial diabetes gene mutation was established by tracing the pedigrees of some positive patients. Family lines 1–4 all conform to matrilineal inheritance, while in family line 5, the proband’s brother (II2) and his two nieces (III1, III2) are negative for the m.3243A>G mutation, which does not conform to matrilineal inheritance (Figure 3).

### 3.4 Reliability evaluation

The detection method used in this study is capable of identifying the m.3243A>G mutation with a mutation load as low as 0.5% in a 0.2 ng DNA background. The detection method was experimentally validated in five clinical trial centers. Compared with first-generation sequencing, the positive concordance rate was 100%, the negative concordance rate was 97.8%, and the overall concordance rate was 98.4%. Additionally, based on the scoring criteria outlined in the expert consensus (中国医师协会检验医师分会线粒体疾病检验医学专家委员会, 2021), all nine patients with a positive m.3243A>G mutation scored ≥ 4 points, representing 100% of the positive cases. Among the 70 negative patients, 51 cases scored less than 4 points, accounting for 71.8%. These findings indicate that the positive patients identified in this study align with the clinical characteristics of a high-risk MDM population, while the majority of negative patients align with the clinical characteristics of a low-risk MDM population. This further validates the reliability of the gene mutation detection method employed in this study.

### 3.5 Satisfaction

We conducted a follow-up survey to assess the satisfaction of patients who had participated in a study involving the collection of oral exfoliated cells for mutation screening. The survey results indicated that all participants experienced no significant discomfort during the procedure. Additionally, a minority of patients reported a mild itching sensation in their throats, but this was generally brief and manageable. Overall, the patients expressed high levels of satisfaction with the method used, with a satisfaction rate reported as 100%.

### 3.6 Compilation of relevant cases from the literature

We collected dozens of case reports related to mitochondrial diabetes mellitus (MDM) and compiled clinical information on 26 individuals with the m.3243A>G mutation who had relatively complete data. After organizing and analyzing this information, we found that 22 patients (84.6%) were under the age of 40, all 26 patients (100%) had a non-obese body type, and all patients (100%) demonstrated maternally inherited mutations. Additionally, 24 patients (92.3%) had hearing impairment, and 19 patients (73.1%) presented with multisystem clinical manifestations, such as migraines, cardiomyopathy, or other systemic complications. Regarding treatment, 21 patients (80.8%) were managed with insulin therapy, while five patients (19.2%) were treated with oral hypoglycemic medications (Table 3).

**TABLE 3:** Case collection in literature.

Patients	m. 3243A>G mutation	Age of onset (years)	BMI(kg/m^2^)	Maternal inheritance	Accompanied by hearing loss	Performance of multiple systems	Lactic acidosis	Diabetic ketoacidosis	Treatment
1	(+)	27	12.49	Y	Y	Y	Y	N	Insulin (Joko et al., 1997)
2	(+)	30	Normal	Y	Y	N	N	N	Insulin (Simões de Carvalho et al., 2023)
3	(+)	32	16.4	Y	Y	Y	Y	N	Insulin (Tabebi et al., 2020)
4	(+)	22	20.2	Y	Y	Y	Y	N	Insulin (Ohwada et al., 2023)
5	(+)	38	23.3	Y	Y	Y	Y	N	Insulin (Bryan et al., 2022)
6	(+)	40	18.6	Y	Y	N	Y	N	Oral (Sakata et al., 2022) medication
7	(+)	27	Normal	Y	Y	Y	Y	N	Oral (Lin et al., 2022) medication
8	(+)	32	16.4	Y	Y	Y	Y	N	Insulin (Tabebi et al., 2020)
9	(+)	41	17.7	Y	N	Y	Y	N	Oral (Oyama et al., 2020) medication
10	(+)	27	21.3	Y	Y	N	Y	Y	Insulin (Sugai et al., 2018)
11	(+)	43	21.0	Y	Y	Y	N	N	Insulin (Alves et al., 2017)
12	(+)	36	19	Y	Y	Y	N	N	Insulin (Alcubilla-Prats et al., 2017)
13	(+)	56	24.1	Y	Y	Y	Y	N	Insulin (Finsterer and Frank, 2015)
14	(+)	13	Normal	Y	Y	Y	Y	N	Insulin (Windpessl et al., 2014)
15	(+)	34	17.0	Y	Y	Y	Y	N	Insulin (Hoptasz et al., 2014)
16	(+)	33	19.0	Y	Y	Y	N	N	Insulin (Naing et al., 2014)
17	(+)	12	Scrubby	Y	Y	Y	N	N	Oral (Cao et al., 2013) medication
18	(+)	19	19.1	Y	Y	N	N	N	Insulin (Mory et al., 2012)
19	(+)	37	Scrubby	Y	Y	Y	Y	N	Insulin (Seidowsky et al., 2013)
20	(+)	24	23.0	Y	Y	N	N	N	Insulin (García et al., 2012)
21	(+)	26	Scrubby	Y	Y	Y	Y	Y	Insulin (Gerber et al., 2010)
22	(+)	15	18.7	Y	Y	N	N	N	Insulin (Zhang et al., 2009)
23	(+)	11	22.1	Y	Y	N	N	N	Insulin (Bergamin et al., 2008)
24	(+)	17	16.3	Y	N	Y	N	N	Insulin (Harihara et al., 2008)
25	(+)	33	22.0	Y	Y	Y	N	N	Insulin (Donovan and Severin, 2006)
26	(+)	32	15.5	Y	Y	Y	N	N	Oral (Chen et al., 2004) medication

## 4 Discussion

Mitochondrial diseases encompass a group of complex disorders that disrupt energy metabolism and can lead to the development of diabetes. Unlike other forms of diabetes, mitochondrial diabetes mellitus (MDM) rarely presents with diabetic ketoacidosis (DKA), which helps differentiate it from other types of monogenic diabetes, including Maturity-Onset Diabetes of the Young (MODY), particularly due to its frequent involvement of multiple organ systems (Yeung et al., 2021). However, MDM often exhibits phenotypic similarities to type 1 or type 2 diabetes, making it prone to being overlooked or misdiagnosed in clinical practice. This has led to an underestimation of its true incidence. This study introduces a novel, non-invasive screening method for mitochondrial diabetes that is convenient, well-tolerated by patients, and reliable. This approach has the potential to become the preferred method for screening mitochondrial diabetes in clinical settings, thereby improving diagnostic accuracy and reducing the incidence of missed or misdiagnosed cases. The incidence of MDM detected using this method among diabetes patients was 3.35%, slightly higher than previously reported. These MDM patients commonly presented with neurological hearing impairment and multisystem clinical manifestations. The genetic pattern in four families was consistent with maternal inheritance, while one family could not be definitively ruled out due to a potentially low mutation burden. In this study, we utilized a qualitative detection method to identify the m.3243A>G mutation. While effective, this approach does not quantify the mutation burden, which limits the scope of the detection method. Upon reviewing literature-reported cases, we observed that patients with the m.3243A>G mutation typically present at a younger age, exhibit a strong maternal inheritance pattern, and frequently experience hearing loss and multisystem involvement. Some cases were associated with lactic acidosis, while diabetic ketoacidosis was almost entirely absent. The primary treatment method was subcutaneous insulin injection. Our findings align closely with these characteristics.

For mitochondrial DNA (mtDNA) mutation screening, blood samples are typically used for analysis in routine clinical settings. However, for large-scale population screening, collecting blood samples is relatively complex, invasive, and costly, making it less practical. Wei and colleagues conducted a rapid screening for the m.3243A>G mutation in 1,070 patients with diabetes and quantitatively assessed the heteroplasmy levels of mutations in six confirmed Mitochondrial Diabetes Mellitus (MDM) patients using pyrosequencing technology. They found that the heteroplasmy levels of the m.3243A>G mutation in urine sediment and oral mucosal exfoliated cells were higher than those in blood samples (Wei et al., 2016). Therefore, mutation detection using oral mucosal exfoliated cells from diabetes patients is more effective than using blood samples, a finding that has been confirmed in previous studies. Additionally, fibroblast growth factor 21 (FGF21) levels increase due to mitochondrial dysfunction. Komorita and other researchers discovered that serum FGF21 levels in MDM patients are significantly higher than in patients with other types of diabetes, suggesting that serum FGF21 could be used for preliminary screening of MDM (Komorita et al., 2023). Early screening and diagnosis of suspected MDM patients can help clarify the underlying pathogenesis and guide clinical treatment strategies.

The molecular mechanism by which the m.3243A>G mutation affects insulin secretion may involve a decrease in cytoplasmic ADP/ATP levels (Maassen et al., 2004). Normal insulin secretion is regulated by potassium channels on pancreatic β-cells. Mitochondrial DNA mutations can lead to increased oxidative stress and damage, as well as dysfunction of the respiratory chain, resulting in reduced adenosine triphosphate (ATP) production and an imbalanced ATP/adenosine diphosphate (ADP) ratio. These changes can disrupt the normal functioning of potassium channels, impairing insulin secretion and potentially leading to insulin deficiency (Maassen et al., 2004; Pang et al., 2001). However, Lindroos et al. reported that individuals with the m.3243A>G mutation exhibit insulin resistance in skeletal muscle, with no significant impairment in beta cell function (Lindroos et al., 2009). Therefore, both insulin deficiency and insulin resistance may contribute to the pathogenesis of mitochondrial diabetes mellitus (MDM).

For patients diagnosed with mitochondrial diabetes mellitus (MDM), promptly and accurately selecting appropriate treatment options can significantly improve prognosis. Due to the inherent mitochondrial mutations in MDM patients and the potential impact of various drugs on mitochondrial function, careful consideration is essential when choosing medications. The use of metformin for MDM treatment may exacerbate mitochondrial dysfunction and lead to adverse effects, such as hyperlipidemia. Therefore, after a diabetes diagnosis, oral metformin should be avoided, and insulin therapy should be initiated as early as possible. Additionally, MDM patients are at high risk for cardiovascular disease mortality. Glucagon-like peptide-1 receptor agonists (GLP-1RA) and sodium-glucose cotransporter-2 inhibitors (SGLT-2i) are cardioprotective agents that may offer beneficial metabolic effects for MDM patients (Yeung et al., 2021). For example, the SGLT-2i Sipagliflozin has been shown to improve the respiratory function of atrial mitochondria (Shao et al., 2019), while the SGLT-2i Dapagliflozin is more effective than insulin in addressing mitochondrial membrane potential dysfunction in cardiomyocytes (Durak et al., 2018).

Before resorting to genetic testing to identify specific mutations, it is feasible to conduct a preliminary assessment of hereditary diabetes based on clinical differences. Sylvie and colleagues found that among patients with diabetes related to rare mitochondrial DNA mutations, the prevalence of nervous system symptoms was higher than in those with the m.3243A>G mutation. In contrast, conditions such as deafness, nephropathy, and macular dystrophy were significantly less common in these patients compared to those with the m.3243A>G mutation (Decoux-Poullot et al., 2020). Additionally, Maturity-Onset Diabetes of the Young (MODY) is an autosomal dominant hereditary diabetes, with glucokinase maturity-onset diabetes of the young (GCK-MODY), caused by glucokinase (GCK) gene defects, being the most common form. GCK-MODY typically presents with mild, asymptomatic fasting hyperglycemia and is rarely accompanied by neurological symptoms (Yeung et al., 2018). This characteristic can help differentiate it clinically from mitochondrial diabetes mellitus (MDM). However, genetic testing remains essential for definitively determining the type of hereditary diabetes.

In summary, this study screened 478 patients with diabetes for mitochondrial diabetes mellitus (MDM) using oral exfoliated cells. Among them, 16 tested positive, including 4 asymptomatic family members. However, only 12 cases were available for clinical statistical analysis, and complete clinical information was obtained for just nine patients. Consequently, the phenotypic statistics may have significant limitations, which could also account for the differences in the positive rate compared to previous studies. Optimizing genetic testing methods and expanding sample sizes are crucial for future research. Additionally, early diagnosis and follow-up treatment of asymptomatic patients are essential for improving their prognosis and quality of life.

## Data Availability

The original contributions presented in the study are included in the article/supplementary material, further inquiries can be directed to the corresponding author.
