# Correction: A non-invasive method for screening mitochondrial diabetes

**DOI:** 10.3389/fgene.2025.1645252

**Published:** 2025-07-08

**Authors:** Hangyu Fang, Xiaoe Li, Shuping Wang, Mei Zhang, Victor Wei Zhang, Chao Xu

**Affiliations:** ^1^ Department of Endocrinology, Shandong Provincial Hospital Affiliated to Shandong First Medical University, Jinan, Shandong, China; ^2^ Key Laboratory of Endocrine Glucose & Lipids Metabolism and Brain Aging, Ministry of Education, Jinan, Shandong, China; ^3^ Department of Endocrinology, Weishan County People’s Hospital, Jining, Shandong, China; ^4^ Department of Endocrinology and Metabolism, Dongying People’s Hospital, Dongying, Shandong, China; ^5^ Affiliated Hospital of Jining Medical University, Jining, Shandong, China; ^6^ Department of Genomic Medicine, AmCare Genomics Lab, Guangzhou, Guangdong, China; ^7^ Shandong Key Laboratory of Endocrinology and Lipid Metabolism, Jinan, Shandong, China; ^8^ Shandong Institute of Endocrine and Metabolic Diseases, Jinan, Shandong, China; ^9^ “Chuangxin China” Innovation Base of Stem Cell and Gene Therapy for Endocrine Metabolic Diseases, Jinan, Shandong, China; ^10^ Shandong Engineering Laboratory of Prevention and Control for Endocrine and Metabolic Diseases, Jinan, Shandong, China; ^11^ Shandong Engineering Research Center of Stem Cell and Gene Therapy for Endocrine and Metabolic Diseases, Jinan, Shandong, China

**Keywords:** mitochondrial diabetes mellitus, m.3243A>G, mutations, screening, non-invasive

In the published article, there was an error in affiliation [1. Key Laboratory of Endocrine Glucose & Lipids Metabolism and Brain Aging, Ministry of Education, Jinan, Shandong, China, 2. Department of Endocrinology, Shandong Provincial Hospital Affiliated to Shandong First Medical University, Jinan, Shandong, China]. Instead of “[1. Key Laboratory of Endocrine Glucose & Lipids Metabolism and Brain Aging, Ministry of Education, Jinan, Shandong, China, 2. Department of Endocrinology, Shandong Provincial Hospital Affiliated to Shandong First Medical University, Jinan, Shandong, China]”, it should be “[1. Department of Endocrinology, Shandong Provincial Hospital Affiliated to Shandong First Medical University, Jinan, Shandong, China, 2. Key Laboratory of Endocrine Glucose & Lipids Metabolism and Brain Aging, Ministry of Education, Jinan, Shandong, China]”.

The authors apologize for this error and state that this does not change the scientific conclusions of the article in any way. The original article has been updated.

